# Assessing Landscape Change and Processes of Recurrence, Replacement, and Recovery in the Southeastern Coastal Plains, USA

**DOI:** 10.1007/s00267-015-0574-1

**Published:** 2015-07-11

**Authors:** Mark A. Drummond, Michael P. Stier, Roger F. Auch, Janis L. Taylor, Glenn E. Griffith, Jodi L. Riegle, David J. Hester, Christopher E. Soulard, Jamie L. McBeth

**Affiliations:** U.S. Geological Survey, Geosciences and Environmental Change Science Center, 2150C Centre Ave, Fort Collins, CO 80526 USA; U.S. Geological Survey, Geosciences and Environmental Change Science Center, Box 25046, DFC MS980, Denver, CO 80225 USA; U.S. Geological Survey, Earth Resources Observation and Science Center (EROS), 47914 252nd St., Sioux Falls, SD 57198 USA; Stinger Ghaffarian Technologies (SGT, Inc.), Contractor to the U.S. Geological Survey (USGS) Earth Resources Observation and Science (EROS) Center, Sioux Falls, SD 57198 USA; U.S. Geological Survey, 200 SW 35th Street, Corvallis, OR 97333 USA; U.S. Geological Survey, Western Geographic Science Center, MS-531, Menlo Park, CA 94025 USA; U.S. Geological Survey, Geosciences and Environmental Change Science Center, 2327 University Way, Suite 2, Bozeman, MT 59715 USA

**Keywords:** Land-use process, Landscape change, Land recovery, Ecoregion, Silviculture, Coastal plain

## Abstract

The processes of landscape change are complex, exhibiting spatial variability as well as linear, cyclical, and reversible characteristics. To better understand the various processes that cause transformation, a data aggregation, validation, and attribution approach was developed and applied to an analysis of the Southeastern Coastal Plains (SECP). The approach integrates information from available national land-use, natural disturbance, and land-cover data to efficiently assess spatially-specific changes and causes. Between 2001 and 2006, the processes of change affected 7.8 % of the SECP but varied across small-scale ecoregions. Processes were placed into a simple conceptual framework to explicitly identify the type and direction of change based on three general characteristics: replacement, recurrence, and recovery. Replacement processes, whereby a land use or cover is supplanted by a new land use, including urbanization and agricultural expansion, accounted for approximately 15 % of the extent of change. Recurrent processes that contribute to cyclical changes in land cover, including forest harvest/replanting and fire, accounted for 83 %. Most forest cover changes were recurrent, while the extents of recurrent silviculture and forest replacement processes such as urbanization far exceeded forest recovery processes. The total extent of landscape recovery, from prior land use to natural or semi-natural vegetation cover, accounted for less than 3 % of change. In a region of complex change, increases in transitory grassland and shrubland covers were caused by large-scale intensive plantation silviculture and small-scale activities including mining reclamation. Explicit identification of the process types and dynamics presented here may improve the understanding of land-cover change and landscape trajectory.

## Introduction

Land-use changes are transforming the biosphere as landscapes and ecological systems become increasingly dominated by anthropogenic processes (Vitousek et al. 1997; Ellis et al. 2010; Ellis 2011). The various human-driven processes of change can be complex, exhibiting spatial variability as well as linear, cyclical, and reversible characteristics (Mertens and Lambin 2000; Rudel et al. 2000; Drummond and Loveland 2010; Watson et al. 2013). Natural disturbances and climate variability and change contribute to the complexity, either directly or indirectly (Marshall et al. 2003; Kates et al. 2012). As a result, the landscape-scale characteristics of land cover can shift markedly over time, requiring periodic critical assessment to understand the causes and processes of change (Lambin 1997; Houet et al. 2010; Hudson and LaFevor 2014).

The diverse processes of landscape-scale change are important to understand but are not always made explicit. Land-cover change analyses typically rely on two or more snapshots of land surface condition that are used to estimate the type, frequency, and magnitude of cover change, whereas it is also informative to explore the land-use causes and characteristic processes of landscape change (Geist and Lambin 2002; Goldewijk and Ramankutty 2004). By incorporating land-use information into the analysis, the extent and implications of human pressures can be better understood (Geldmann et al. 2014). Because of the diversity of underlying biophysical and socioeconomic factors interacting across the terrestrial biosphere, anthropogenic processes and their effects on land cover likely exhibit substantial variability at the smaller landscape scale. An improved landscape-scale understanding of how processes vary across regions, such as explored here, may provide important insight for management and policy efforts concerning conservation and global change issues (Millard et al. 2012).

**Fig. 1:**
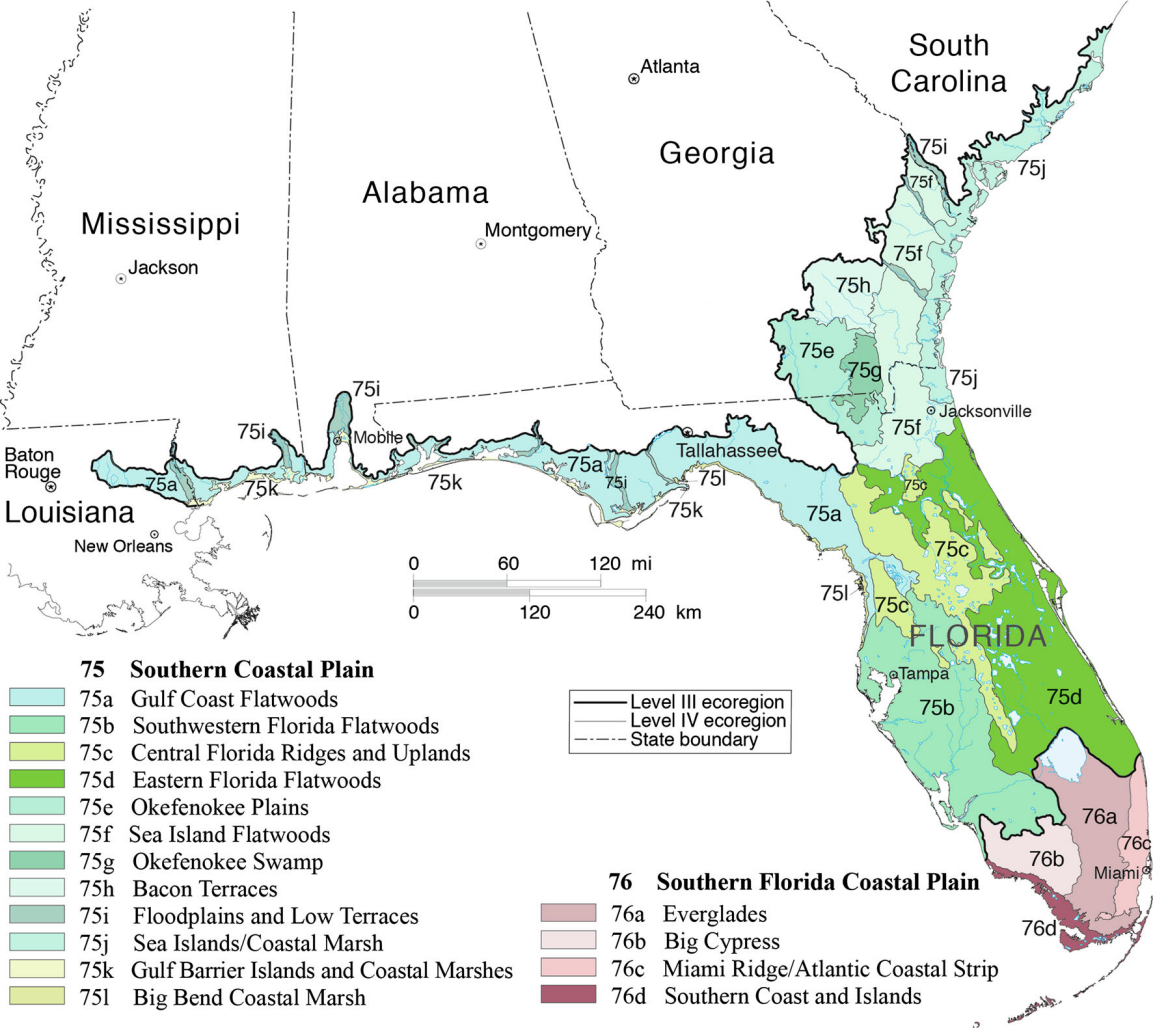
Map of the SECP study area showing level III and IV ecoregions (USEPA 2013)

New approaches are needed to improve the understanding of human influence across broad spatial scales (Sanderson et al. 2002; Woolmer et al. 2008; Verburg et al. 2013). At the same time, there is a need to develop a more complete understanding of the different landscape-change processes beyond a strict focus on the gains and losses among major land-cover types (Velázquez et al. 2003; Lasanta and Vicente-Serrano 2012; Emili and Greene 2014). Here, we work toward these goals by developing and implementing an approach to investigate proximate land-use causes and natural disturbances across 16 landscape-scale ecoregions (USEPA 2013) within the high-change Southeastern Coastal Plains region (SECP; Fig. 1). The approach incorporates information from available national datasets and is designed to provide consistent and comparable information on the landscape changes occurring across large regions of the US. The proximate causes identified by the study are placed into a simple conceptual framework to explicitly identify the type and directional characteristics of change including (1) land-use and land-cover changes that are recurring, (2) simple replacement of land cover by another cover type, and (3) recovery of semi-natural or secondary land cover from a prior land use.

Two additional objectives related to land-use dynamics are also prominent. The first objective is to understand the recurrent land-cover dynamics of intensive silviculture. Intensively managed plantation silviculture is prevalent throughout the southeastern US and other world regions (Zhang and Polyakov 2010). However, there is not a clear approach for incorporating the dynamics of recurrent harvest and reforestation into land change analyses. The second objective is to understand the extent and origin of land that transitions out of intensive land use into semi-natural recovery. Although eastern forests have recovered via historical processes that are well-examined by forest transition theory (Mather and Needle 1998; Rudel et al. 2005; Barbier et al. 2010), forest cover persistence or further expansion may be limited by recent anthropogenic pressures on land resources (Drummond and Loveland 2010; Jeon et al. 2014). For the analysis, several national sources of land change data are combined, validated, and further attributed to develop the approach for systematic landscape-scale assessment across large areas.

## Methodology

### Study Area

The SECP study area covers approximately 16.4 m ha and two regional-scale EPA level III ecoregions, the Southern Coastal Plain (ecoregion 75) and the Southern Florida Coastal Plain (ecoregion 76). It includes much of Florida and small parts of Georgia, South Carolina, Alabama, Mississippi, and Louisiana. The area has a mild mid-latitude humid subtropical climate, marked by hot, humid summers and warm to mild winters. The Southern Florida Coastal Plain is nearly frost free, with more of a tropical savanna climate. Mean annual temperatures for the SECP are 20–25 °C, and annual precipitation ranges from 1170 to 1650 mm (Griffith et al. 1994). The flat, alluvial plains, and marine terraces are composed mostly of sands and gravels, along with silt, clay, peat, and muck, and are underlain in places by limestone. Elevations range from sea-level to 88 m. Low-gradient streams and rivers occur, along with numerous wetlands, and more than 7000 lakes (Wiken et al. 2011).

The SECP is a region of complex land-use and land-cover change, which provides a suitable area to explore processes of recurrence and recovery. The region is characterized by high population growth, a tourism-dependent economy, and intensive land uses including pine plantations and high-value agriculture such as sugar cane and citrus (Walker 2001; Kambly and Moreland 2009; Drummond 2015). Substantial areas in the south, including at least half of the Everglades region with sawgrass (*Cladium jamaicense*) marshes, sloughs, and hardwood hammocks, have been affected by land-cover changes and historical efforts to re-engineer the wetlands as well as recent efforts to restore ecological function (Marshall et al. 2003; Walker and Solecki 2004; Hogan et al. 2012). The growth of commercial pine plantation silviculture in the north has played a significant role in replacing agriculture and more natural forested ecosystems, although plantation forests are also an important renewable source of wood and fiber (Wear et al. 2007; Napton et al. 2010; Oliver et al. 2014). A suite of landscape-change processes have substantial effects on the extent of native longleaf pine forests (*Pinus palustris*), wetland dynamics, climate, carbon flux, and coastal ecosystems (Binford et al. 2006; Mitchell and Duncan 2009; Pan et al. 2013; Zhao et al. 2013; Trail et al. 2013). Interactions with a changing climate may magnify the increasing pressures from land use (Twilley et al. 2001).

### Overview of Approach

A flexible data aggregation, validation, and attribution (AVA) approach was developed for this assessment of landscape change that involved combining available sources of spatial data to (1) create a refined spatially explicit analysis of landscape change and (2) facilitate the identification of proximate land-use and natural-disturbance causes. Spatial data including land-use and land-cover maps, digital orthoimagery, and satellite imagery are increasingly available at the national-level and at multiple time steps. To take advantage of this accessibility, we examined landscape change by combining several thematic land-use, land-cover, and disturbance data available for the conterminous US. The approach is portable and adaptable to other US regions. As part of the analysis, the change data were further validated with high-resolution imagery and attributed with the proximate cause of change using a combination of spatial analysis, decision trees, and manual verification. Estimates were then compiled at the landscape scale using USEPA level IV ecoregions, the smallest land unit identified in the multi-scale ecoregion dataset (USEPA 2013; Omernik and Griffith 2014). The approach is flexible, such that new data may be added as they become available and decision rules can be modified as other regions with different land-use and disturbance dynamics are examined.

The basic techniques employed here are in common practice but do not have an extensive history of use for landscape-change assessment. However, pre-existing thematic maps have been used to improve the quality of new satellite-derived land-cover characterizations (Stewart 1998); to develop maps representative of human influence (Sanderson et al. 2002; Leu et al. 2008; Woolmer et al. 2008); and to create a land-use map using land cover as the underlying structure (Theobald 2014). The specific land use in a given location can sometimes be inferred directly from the land cover; otherwise, additional sources of information can be combined to facilitate the identification of the land use (Batista e Silva et al. 2013). Our approach creates separate co-registered land-use change and land-cover layers.

### Landscape-Change Analysis Process

**Fig. 2:**
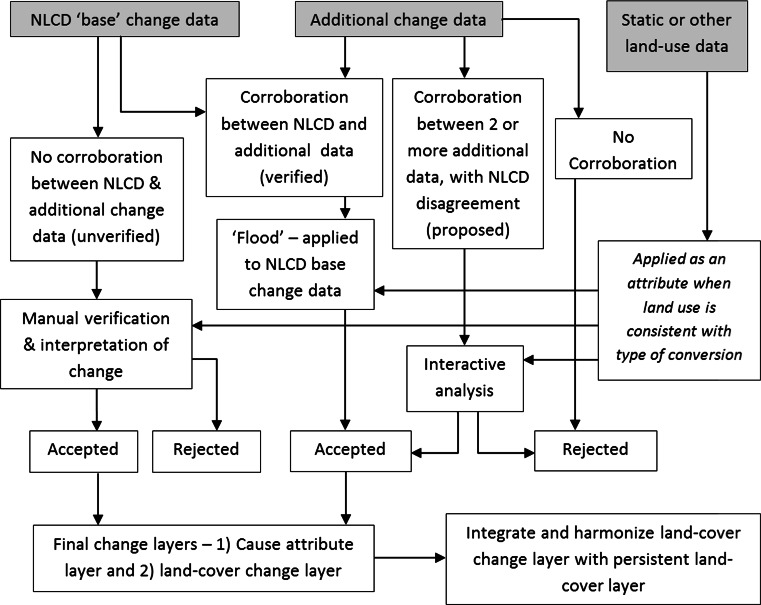
Overview of the AVA processing steps used in the analysis

Suitable national-scale data of land use, land cover, and natural disturbance were identified for aggregation and further analysis (Table 1). The focus was on data available across the conterminous US in order to develop a consistent approach useful for national land change assessment. The sources were primarily thematic raster but also polygon and point data. The 30-m resolution multi-date National Land Cover Database (NLCD) provided a comprehensive base land-cover change dataset (Homer et al. 2007; Fry et al. 2011). The NLCD has complete coverage of the conterminous US for major land-cover types at a suitable resolution, whereas the other datasets generally focus on a limited set of land-cover or land-use occurrences. An NLCD change map was created from the 2001 and 2006 NLCD by computing the difference between the two raster images, which provided a preliminary change layer coded with from/to conversions. The preliminary, or base, change layer was further augmented with additional change information during the AVA process (Fig. 2).

**Table 1:** Principal data used in the analysis

Dataset	Dates used	References
1. National Land Cover Dataset (NLCD)	2001, 2006	Homer et al. (2007) and Fry et al. (2011)
2. Mining, from the 1992 NLCD	1992	Vogelmann et al. (2001)
3. Monitoring Trends in Burn Severity (MTBS)	1999–2006, annual	Eidenshink et al. (2007)
4. Hansen/UMD/Google/USGS/NASA Global Forest Change 2000–2012	2000–2012, including annual	Hansen et al. (2013)
5. Vegetation Change Tracker (VCT)	1999–2006, annual	Huang et al. (2009)
6. Landfire Disturbance	1999–2006, annual	Landfire (2013)
7. Mineral Resources Data System (MRDS)	2012	US Geological Survey (2005)
8. Forest Ownership in the Conterminous United States	2007	Nelson et al. (2010)
9. Census Urban Area	2000, 2010	US Census Bureau (2011)

After identifying the relevant spatial data and preparing the preliminary landscape-change layer from the NLCD, an algorithm written in C++ using the GDAL libraries was developed to automatically generate a single intermediate landscape-change map from the various data sources. The AVA approach developed for this study relies on the algorithm for analysis of the level of corroboration among the various data as well as on manual interpretation techniques using higher resolution digital imagery. The AVA algorithm specifically uses spatial data comparison and a decision tree process to identify and label 30-m raster pixels in the intermediate layer as either a verified change (NLCD is corroborated), a proposed change (corroborated without NLCD) that needs further verification or an unverified change (NLCD is not corroborated) that needs manual interpretation. When the various input data have relevant land-use information, it is also transferred to the intermediate layer as a potential cause attribute. When pixels from non-NLCD sources indicate a potential change, but there is no corroboration with the NLCD or any other data, the information is saved to a separate auxiliary file. After visual inspection, the auxiliary file was rejected for use because the uncorroborated ‘change’ occurred outside the temporal range of the study or did not represent a true landscape change (noise). The algorithm also uses data sources prior to 2001. For example, natural disturbance and forest harvest information that pre-dates 2001 is used to help identify prior events that led to, for example, forest regrowth during the study period. If a potential change site was ultimately determined to have no change, it was revised to the appropriate land cover to indicate persistence during both dates.

As noted above, a verified change occurs when an NLCD conversion is corroborated by additional data. However, the verification depends on the specific type of NLCD from/to conversion. For example, if the additional data show a mechanical clearance of forest (including the ‘clearcut’ and ‘harvest’ classes from the Landfire Disturbance data listed in Table 1) and the intersecting NLCD change layer shows a spatially corresponding conversion from forest to a herbaceous cover type, then the change parcel is labeled as a verified forest change in the intermediate change layer because the NLCD base layer and at least one other data source are in agreement. In this case, the recently harvested area was classified as a change from forest cover in 2001 to a non-forest cover in 2006, such as herbaceous grassland. The interpretation was checked against other spatial data for conflicting associations that would require an extra manual verification step to resolve the confusion and determine the change, which eliminates other possible causes.

Since landscape patterns from different raster datasets rarely have precise spatial alignment, which is common for land-cover maps derived from different satellites, resolutions, time of season, or software, the AVA algorithm uses the NLCD to define the spatial pattern of change. For example, where at least two raster datasets identify that a landscape change occurred at a specific location, there are also adjacent pixels from each dataset that do not spatially align with each other. In this case, the adjacent pixels from the NLCD are coded (flooded) with the same landscape change class as the area where the datasets agree. The adjacent pixels from the other datasets are rejected as noise. In this case, the use of the NLCD as the base layer allowed for spatial consistency when the different datasets did not exactly align.

Once the intermediate change layer was completed, pixel groups that were labeled as a proposed change (corroborated without NLCD) were interactively assessed at randomly selected sites using higher resolution digital imagery to determine the suitability for incorporation into the final change layers. Unverified changes (NLCD is not corroborated) underwent intensive manual interpretation. The primary data source for manual interpretation of landscape change was historical Google Earth imagery (GE, http://earth.google.com). The GE database was particularly useful for examination of dates that are outside of the 2001–2006 period in order to help determine the longer temporal process of change. Ancillary high-resolution digital orthoimagery from the National Agricultural Imagery Program (NAIP) and Landsat satellite data supplemented the analysis as needed. Manual interpretations were aided by air photo interpretation techniques and visual cues such as rows of planted trees that indicate plantation forests, newly exposed bare ground surrounding a fluctuating water body, signs of haying activity, or areas of leveled trees with a date and location that corresponds with the main path of a major hurricane. Other ancillary datasets were used to aid in land-use or land-cover interpretations including US Department of Agriculture (USDA) Forest Types of the United States (Ruefenacht et al. 2008), the US Geological Survey (USGS) The National Map (Sugarbaker and Carswell 2011), and the National Wetlands Inventory (USFWS 2014).

The final output from the AVA approach created three new 30-m resolution landscape change layers, including the 2001 and 2006 land-cover layers and a proximate cause layer, that were created using a mixture of automated (mutual data agreement) and manual techniques. A modified NLCD classification system (Fry et al. 2011; http://www.mrlc.gov/nlcd06_leg.php) was used for the final land-cover layers, in which the four NLCD (urban) developed classes were combined into one urban/developed class and the deciduous and mixed forest categories were combined into a deciduous/mixed forest class. To create two complete wall-to-wall land-cover maps for the region, we merged our 2001 and 2006 change layers with the remaining area of persistent land cover from the NLCD. The final proximate cause layer differs from the land-cover maps because it is classified based on the land use or natural disturbance cause of change.

In this paper, new urbanization was classified as either ‘urban area infill’ or ‘urban expansion.’ Urban Area cartographic boundaries from the 2000 and 2010 Census were used to develop the classification (US Census Bureau 2011). The 2000 Urban Area boundary from the Census, modified for this study, defines the area of infill. However, because the newer 2010 Census used higher resolution census units (blocks) to define Urban Area boundaries, we retroactively removed extraneous non-urban areas from the 2000 data by eliminating any part that extended outside the more-refined 2010 boundary, with the assumption that a non-urban location from 2010 was also not an urban location in 2000. New urbanization and development that occurred outside of the modified urban boundary was classified as an urban expansion.

### Error Assessment

Rigorous approaches for evaluating land-use and land-cover change interpretation errors include random sampling based on a classification of ‘change’ and ‘no change’ (Fuller et al. 2003; Yuan et al. 2005). Here, reference data were aggregated for an accuracy assessment of the ‘change’–‘no change’ classification at 465 randomly selected points. The land-cover classes for 2001 and 2006 were collected for each point location using manual interpretation of historical high-resolution imagery in Google Earth supplemented with additional orthoimagery at the discretion of the analyst. If a clear interpretation could not be made because the point fell on a boundary between land-cover types, the point was rejected. Six sample points were rejected. The reference information that was collected was compared against the new 2001 and 2006 land-cover change layers and the existing persistent (no change between 2001 and 2006) NLCD data. An error matrix was constructed to assess the accuracy of the classification.

## Results and Discussion

### Processes of Change in the SECP

**Fig. 3:**
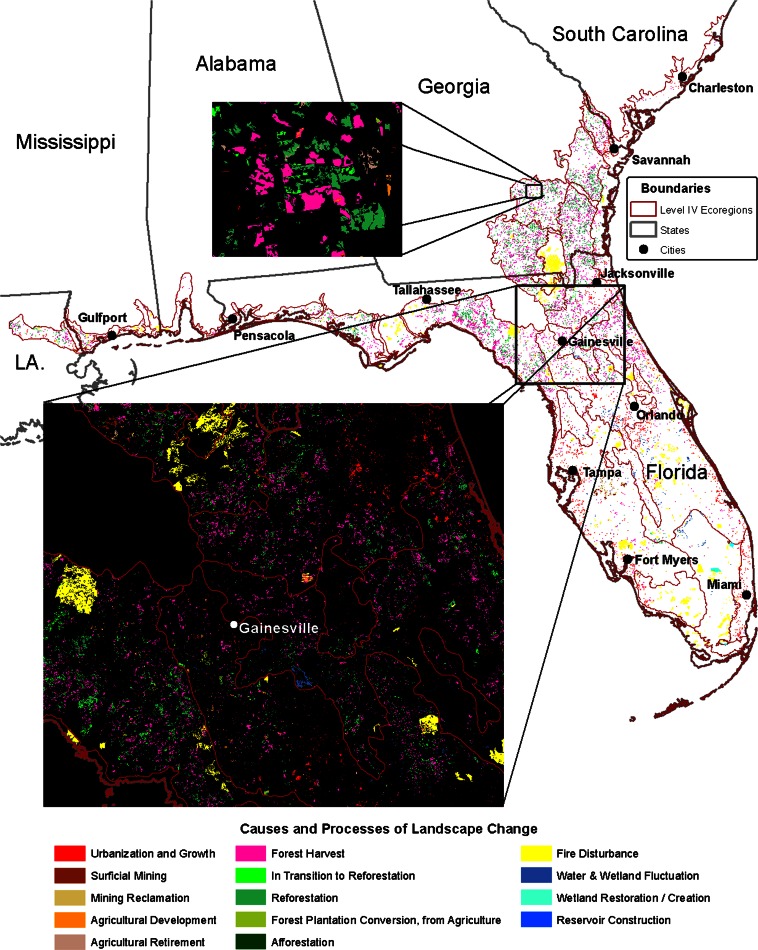
The main causes and processes of landscape change in the SECP between 2001 and 2006, consolidated from Table 2. Categories have been consolidated for viewing at the regional scale

The diverse proximate causes of landscape change between 2001 and 2006 were identified for the SECP and categorized into 12 types of processes (Table 2). The spatial pattern of many of the main causes of landscape change is depicted in Fig. 3. The total area affected by these processes was 1,283,760 ha, which is 7.8 % of the study area. Conceptually, the types of processes are grouped according to three main characteristics: simple land-use/cover replacement, recurrent processes caused by cyclic land-use practices or natural disturbances, and landscape recovery from prior land use.

**Table 2:** A summary of the proximate causes and processes of change in the SECP region from 2001 to 2006, in hectares

Proximate cause	Total (ha)	Type of process	Total process (ha)
*Replacement*			
Urban expansion (residential, commercial, industrial)	88,001	Urbanization and growth	140,730
Residential ponds	(458)		
Urban area infill (residential, commercial, industrial)	52,729		
Residential ponds	(120)		
Cropland conversion	14,513	Agricultural development	21,936
Pasture/hay conversion	7423		
Forest plantation conversion from agriculture	4244	Intensive silviculture expansion	4244
Surficial mining	19,728	Mining and energy extraction	19,728
Oil/gas pads	0		
Reservoir construction	1772	Surface water management	1908
Flood control, including levees	136		
Total replacement			188,546
*Recurrence*			
Forest harvest (including wetland forest)	427,067	Intensive silviculture and other timber extraction	747,882
Reforestation	243,076		
In transition to reforestation	77,739		
Lake and reservoir-level fluctuation	20,848	Water and wetland flux	24,650
Wetland fluctuation	1640		
Sea-level/beach fluctuation	1662		
Stream fluctuation	304		
Flooded agriculture	194		
Forest fire, stand loss	4876	Fire disturbance	292,525
Forest regrowth after fire	11		
Other natural/human-caused fire, no stand loss	287,638		
Windthrow	373	Other natural disturbance	375
Sand dune movement, following storms	2		
Total recurrence			1,065,432
*Recovery*			
Wetland restoration/creation	10,483	Recapture ecosystem service	19,142
Mining reclamation	8660		
Cropland abandoned, revegetation in progress	8668	Agricultural retirement	9567
Pasture abandoned, revegetation in progress	899		
New forestation, de-intensification of land use	1073	Afforestation	1073
Total recovery			29,782
Total for SECP			1,283,760

Processes are grouped by three major characteristics: replacement of previous land use or land cover with a new land use; recurrent processes that are cyclical or frequent in nature; and recovery of the landscape through reclamation, restoration, and land-use retirement. Numbers shown in parentheses are sub-categories that are already included in the total for the associated proximate cause

Replacement of a previous land use or land cover with a new land use is caused primarily by urban expansion and infill (140,730 ha), conversion to cropland and pasture/hay (21,936 ha), forest plantation conversion from agriculture (4244 ha), surficial mining (19,728 ha), and water management including reservoir construction (1908 ha). Approximately 37.5 % of the new urbanization in the SECP occurred as infill, while 62.5 % occurred as expansion beyond the modified 2000 Census Urban Area boundary. Although agricultural land use (cropland and pasture/hay) had some localized gains, it lost ground overall to urbanization and other land uses. In areas where agriculture is replaced by plantation forest (4244 ha), it may be harvested and put into a recurrent cycle in the future. Approximately 14.7 % (188,546 ha) of the total SECP processes were caused by replacement.

Recurrent changes are caused primarily by plantation silviculture practices that have a cyclic component, with its temporal pattern of forest harvest and reforestation (747,882 ha). Natural disturbance is most prevalent as vegetation fluctuation caused by fire (292,525 ha). Most fire disturbance did not cause a prolonged, or stand-replacing, change in vegetation. Areas of major fire disturbance that resulted in stand-destruction (4876 ha) are assumed to transition to an initial seral vegetation stage, whereas typical regrowth of the potential vegetation type is a longer process. Recurrent changes also occur when water (lake, reservoir, sea, and stream) and wetland levels fluctuate due to drawdown, agricultural practices, or natural variation in precipitation (24,650 ha), and as windthrow and dune movement overtakes vegetation (375 ha). Approximately 83 % (1,065,432 ha) of the total area of change was affected by recurrent processes.

Recovery occurs as a restoration, creation, reclamation, or retirement of a previous land use to a natural or semi-natural vegetation cover such as occurs with wetland restoration (10,483 ha). Policies and initiatives that encourage land conservation or that require reclamation may drive recovery, including reclaimed mine lands (8660 ha). It also occurs after land use is abandoned or retired (9567 ha), or has had sufficient time to grow to secondary forest (1073 ha). Recovery of a semi-natural land cover, such as identified here, does not necessarily infer that former ecological conditions or functions are recovered. Only 2.3 % (29,782 ha) of the total area was affected by processes of recovery.

**Fig. 4:**
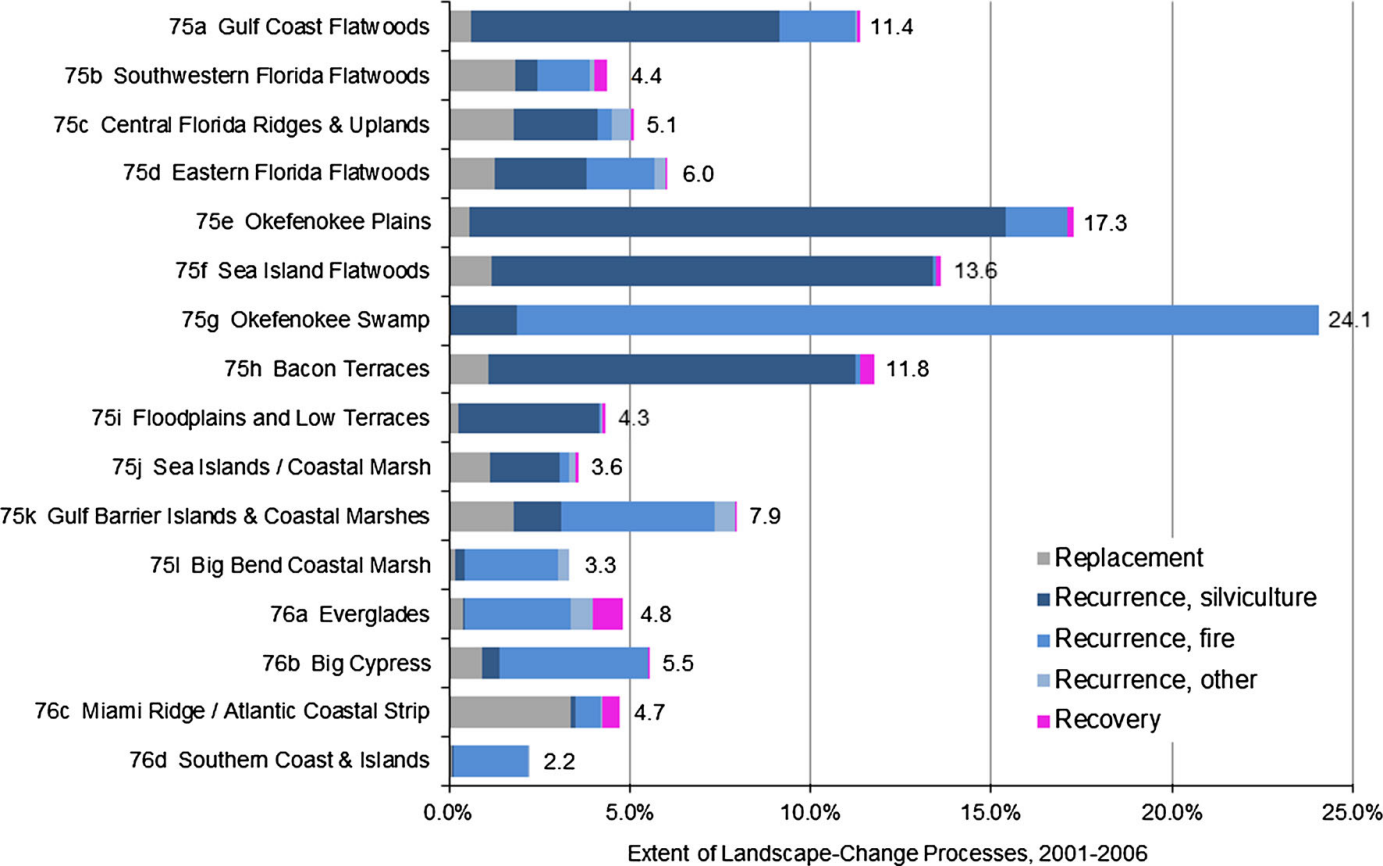
Comparison of the extent of replacement, recurrence, and recovery processes at the landscape-scale (expressed as a percent of Level IV ecoregion area)

The composition and magnitude of SECP processes vary across the 16 landscape-scale ecoregions (Fig. 4), illustrating the differential importance of recovery versus replacement versus recurrent processes. The total extent affected by the various processes ranges from a low of 2.2 % for the Southern Coast and Islands in the south to a high of 24.1 % for the Okefenokee Swamp in the north. In general, recurrent processes are prominent throughout the SECP, although changes caused by plantation silviculture activities occur primarily in the more-northern ecoregions. Recurrence is the most extensive type of change in most ecoregions, whereas replacement is most extensive in only one ecoregion (Miami Ridge/Atlantic Coastal Strip, hereinafter Miami Ridge). Recovery from prior land use is generally small in extent, at less than 1 percent, and did not occur in every ecoregion.

The relative importance of processes of recovery, in relation to replacement and recurrence, is well below 3.5 % in most ecoregions (Table 3). However, recovery is highest in the Everglades (17.4 %) where there is a concerted effort to repair hydrology and habitat, including the conversion of agricultural land to restored wetland (Kambly and Moreland 2009). The Southwestern Florida Flatwoods (8.2 %) and Miami Ridge (9.8 %) are also above 3.5 %. Recurrent processes comprise more than half of the total amount of landscape change in all but two ecoregions (Southwestern Florida Flatwoods, 49.6 %; Miami Ridge, 19.1 %). Replacement processes comprise less than half the total amount in all ecoregions except the highly urbanized Miami Ridge (71.2 %).

**Table 3:** Relative importance of processes of replacement, recurrence, and recovery, in percent

Ecoregions	Replacement	Recurrence	Recovery
75A Gulf Coast Flatwoods	5.5	93.8	0.7
75B Southwestern Florida Flatwoods	42.2	49.6	8.2
75C Central Florida Ridges & Uplands	34.9	63.3	1.8
75D Eastern Florida Flatwoods	21.0	78.5	0.5
75E Okefenokee Plains	3.3	95.9	0.9
75F Sea Island Flatwoods	8.6	90.6	0.8
75G Okefenokee Swamp	0.2	99.8	0.0
75H Bacon Terraces	9.1	87.4	3.4
75I Floodplains and Low Terraces	5.8	92.4	1.8
75J Sea Islands/Coastal Marsh	31.9	65.7	2.4
75K Gulf Barrier Islands & Coastal Marshes	22.8	77.2	0.1
75L Big Bend Coastal Marsh	5.8	94.2	0.0
76A Everglades	8.2	74.4	17.4
76B Big Cypress	17.0	82.9	0.2
76C Miami Ridge/Atlantic Coastal Strip	71.2	19.1	9.8
76D Southern Coast & Islands	3.9	96.1	0.0

### Land-Cover Change in the SECP

#### Land-Cover Accuracy

For each of the two major categories (change, no change), a minimum of 200 sample points were examined to assess the accuracy of the landscape change information. A total of 200 points were examined in areas of ‘change’ and 259 points in areas of ‘no change’ (Table 4). The overall accuracy based on the two categories was 96.5 %. Areas of ‘change’ had a user’s accuracy of 97.5 % and a producer’s accuracy of 94.7 %. Areas of ‘no change’ had a user’s accuracy of 95.8 % and a producer’s accuracy of 98.0 %.

**Table 4:** Error matrix for areas of change and no change in the SECP

Classification	Change	No change	Total	User’s accuracy (%)
Change	195	5	200	97.5
No change	11	248	259	95.8
Total	206	253	459	
Producer’s accuracy	94.7 %	98.0 %		
Overall accuracy				96.5

The study was focused on identifying the areas of change; however, a provisional regional class-by-class land-cover accuracy assessment for 2001 and 2006 was calculated based on spatial integration of the ‘change’ data with the existing ‘no change’ NLCD (Table 5). Accuracies were calculated for the two categories separately based on percent area sampled and then added together to get the overall accuracy for the region. Overall accuracies for land-cover classes with at least 20 samples range from a high of 100 % (2001 and 2006) for water and approximately 95 % (2001 and 2006) for evergreen forest to a low of approximately 63 % (2001 and 2006) for grassland and 69 % (2001) and 64 % (2006) for shrubland. However, grassland accuracies were greater than 95 % and shrubland accuracies were greater than 85 % for the change assessment alone.

**Table 5:** User’s accuracy for 11 land-cover categories

Class	Change assessment					Persistent land cover					Regional totals			
	2001 samples		2006 samples		% area sampled	2001 samples		2006 samples		% area sampled	2001 samples		2006 samples	
	#	Accuracy	#	Accuracy		#	Accuracy	#	Accuracy		#	Overall accuracy	#	Overall accuracy
Evergreen forest	91	95.6	36	94.4	7.8	43	95.3	42	95.2	92.2	134	95.4	78	95.2
Woody wetland	15	93.3	11	54.5	7.8	71	88.3	79	92.4	92.2	86	88.7	90	89.5
Urban/developed	13	100.0	23	95.7	7.8	38	89.2	37	91.9	92.2	51	90.0	60	92.2
Shrubland	33	90.9	41	85.4	7.8	15	66.7	16	62.5	92.2	48	68.6	56	64.3
Herbaceous wetland	8	62.5	5	60.0	7.8	26	84.6	26	84.6	92.2	34	82.9	31	82.7
Cropland	6	83.3	1	100.0	7.8	25	92.0	25	92.0	92.2	31	91.3	26	92.6
Grassland	21	95.2	59	98.3	7.8	5	60.0	5	60.0	92.2	26	62.7	64	63.0
Pasture/hay	4	75.0	1	100.0	7.8	19	89.5	19	78.9	92.2	23	88.3	20	80.6
Water	5	100.0	12	100.0	7.8	16	100.0	9	100.0	92.2	21	100.0	22	100.0
Bare	3	100.0	11	81.8	7.8	0	–	0	–	92.2	3	–	11	–
Decid/mix forest	1	0.0	0		7.8	1	100.0	1	100.0	92.2	2	–	1	–

Overall accuracy was not calculated for classes with less than 20 total samples

#### Land-Cover Change, 2001–2006

The total extent of SECP land-cover change between 2001 and 2006 is 969,222 ha, which is 5.9 % of the SECP (Table 6). The extent of land-cover change is less than the total extent of land-use and disturbance processes discussed above in Table 2 (7.8 % of the SECP) because two processes related to urban intensification and low-intensity fire disturbance caused a modification rather than a strict change in land cover. Urban intensification (0.1 % of the SECP), which is an urban (open space) to urban (higher density) modification of the existing cover type, was not included as a land-cover change. As well, lower-intensity fire disturbances that did not markedly destroy forest or other land covers (1.8 % of the SECP) are not represented here as a strict land-cover change. Forest fires with stand loss, shown in Table 2 (0.03 % of the SECP), are included here as a land-cover change.

**Table 6:** Gross and net land-cover change between 2001 and 2006, in hectares

Land cover	Gross gain	Gross loss	Gross total	Net change	Ratio of absolute net change to gross total	Net change, based on SECP extent (%)	Sector change, based on 2001 land cover (%)
Shrubland	198,913	168,698	367,612	30,215	0.08	0.18	2.84
Herbaceous wetland	49,118	39,315	88,433	9803	0.11	0.06	0.50
Bare	24,701	16,833	41,534	7867	0.19	0.05	9.17
Grassland	279,717	160,563	440,281	119,154	0.27	0.73	27.56
Evergreen forest	229,956	404,503	634,459	−174,547	0.28	−1.06	−6.18
Cropland	14,516	37,562	52,078	−23,046	0.44	−0.14	−2.03
Pasture/hay	7636	27,148	34,785	−19,512	0.56	−0.12	−1.53
Woody wetland	19,354	104,054	123,409	−84,700	0.69	−0.52	−1.84
Water	34,937	2962	37,899	31,976	0.84	0.19	4.64
Deciduous/mixed forest	291	7405	7696	−7114	0.92	−0.04	−6.19
Urban/developed	110,082	177	110,259	109,904	1	0.67	5.05
Total extent of change	969,222						
Absolute net change				617,838			

The total extent of change can be calculated from either all gross gains or all gross losses

The total extent of SECP land-cover change is approximately 350,000 ha greater than the absolute amount of net land-cover change (617,838 ha). This occurs because all land-cover classes had gross (total) gains as well as gross losses that did not result in a net change (Table 6). Net change is the difference between gross gain and gross loss. The net change ratio in Table 6 shows the amount of absolute net change relative to the total amount of gross change for each land-cover class, and indicates large differences in many classes. A ratio near 0 indicates that the amounts of gross gain and loss are nearly equal, resulting in a large magnitude of total change compared to low net change. Classes with lower ratios of 0.08 through 0.28 (shrubland, herbaceous wetland, bare, grassland, and evergreen forest) are closely associated with forest harvest and reforestation activities. Forest areas that are harvested result in transitory bare, herbaceous (grassland or wetland), and shrubland covers. However, other conversions also contribute to the changes in these classes, such as loss to urbanization. Higher ratios have a greater magnitude of either gross gain or loss that result in more similar amounts of total gross change and absolute net change for that class. Urban/developed has a ratio of 1.0 because it is primarily a unidirectional change.

Total gross change was largest for evergreen forest (634,459 ha), grassland (440,281 ha), and shrubland (367,612 ha), which are tied primarily to timber harvest/replanting activities and urbanization. The amount of net change was largest for evergreen forest (−174,547 ha; −1.06 % of SECP), grassland (119,154 ha; 0.73 % of SECP), urban/developed (109,904 ha; 0.67 % of SECP), and woody wetland (−84,700 ha; −0.52 % of SECP). Net increases occurred for shrubland (30,215 ha), herbaceous wetland (9803 ha), bare land (7867 ha), grassland, water (31,976 ha), and urban/developed land cover. Net decreases occurred for evergreen forest, cropland (−23,046 ha), pasture/hay (−19,512), woody wetland, and deciduous/mixed forest cover (−7114 ha).

Similar regional-scale trends in land cover are reported by the sample-based National Resources Inventory (NRI) for the state of Florida from between 2002 and 2007 (USDA 2009). The NRI estimates show increases for water (15,135 ha) and developed land (230,469 ha), while decreases occurred for cropland (−32,982 ha) and forest land (−35,815 ha). The results shown in Table 6 for comparable classes from this study are within the NRI’s margin of error. The NRI estimates are based on non-federal land at the state-level, which likely accounts for some of the difference in the magnitudes of change between the two studies. For example, a substantial amount of forest cover change from this study occurs outside of Florida, in southeastern Georgia, and on federal land. Some of the variation could also occur due to differences in the classification systems or differences between the wall-to-wall approach of this study and the NRI sampling approach. Another sample-based study estimated approximately 110,000 ha of forest cover decrease across the SECP during an 8-year period from 1992 to 2000 (Drummond 2015). Although the 1992–2000 estimate indicates that a substantial amount of annual forest cover decrease occurred (~13,750 ha), it is still less than half the 2001–2006 average annual extent (34,909 ha). Since the extent of net forest cover change depends in part on the rate of forest harvest versus regrowth, substantial temporal differences in the rate of change could have occurred as timber demand or other conditions varied.

**Fig. 5:**
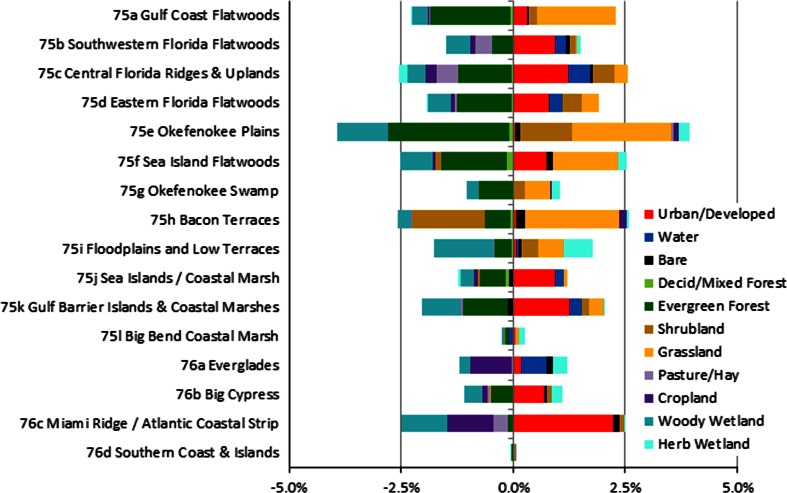
Net land-use and land-cover change in the landscape-scale level 4 ecoregions between 2001 and 2006, based on ecoregion area

Although the net land-cover changes from this study are distributed unevenly across the level 4 ecoregions (Fig. 5), as would be expected with differential patterns of population, natural resource extraction, and land protection, there are some commonalities. All ecoregions with forest cover had a decrease in forest cover. The same is true for woody wetland. Cropland and pasture/hay primarily declined, except for moderate net increases in the Okefenokee Plains and cropland expansion in the Bacon Terraces. Several ecoregions had small gains in herbaceous wetland that are most often tied to woody wetland clearance. Nearly all ecoregions had a net gain in surface water.

### Dynamics of Landscape Change

The dynamics of change, including proximate causes, driving forces, and land-cover changes, are dominated by silviculture and urbanization at the regional scale and across many of the individual ecoregions. However, there is also spatial variability inherent in these processes and important nuances in the characteristics and drivers of change, including forest dynamics and landscape recovery.

#### Urbanization and Replacement Processes

**Fig. 6:**
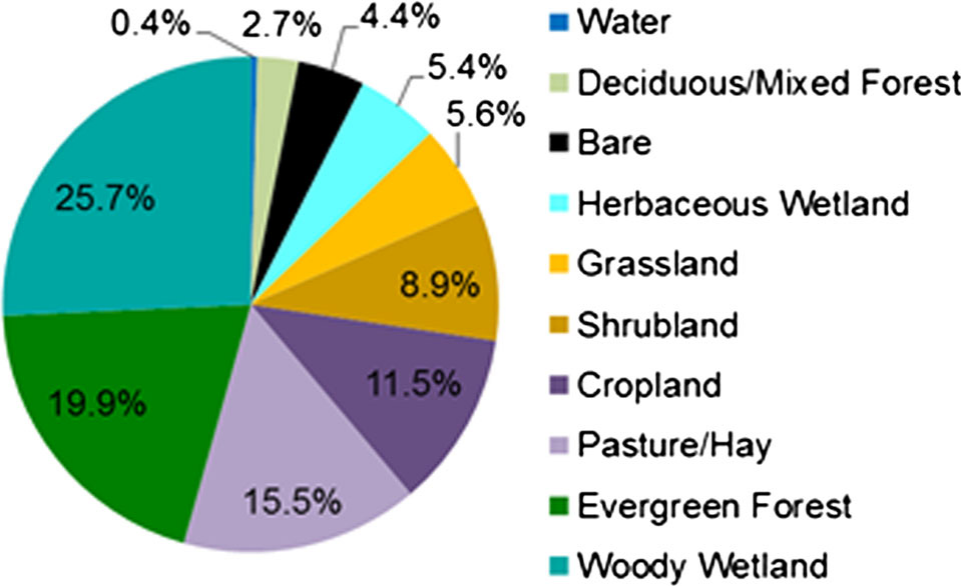
Sources of new urban growth in the SECP between 2001 and 2006

The extent of replacement processes at the landscape scale, expressed as a percent of EPA level IV ecoregion area, vary from more than 3 % (Miami Ridge) to a low of 0 % (Okefenokee Swamp). Region-wide, urbanization is the primary cause of land-cover replacement. New urban/developed land cover, not including processes of urban intensification, increased at an annual rate of 0.13 % (21,980 ha). Woody wetland (25.7 %), evergreen forest (19.9 %), and agricultural lands (pasture/hay, 15.5 %; cropland, 11.5 %) were substantial sources of urban growth (Fig. 6).

**Fig. 7:**
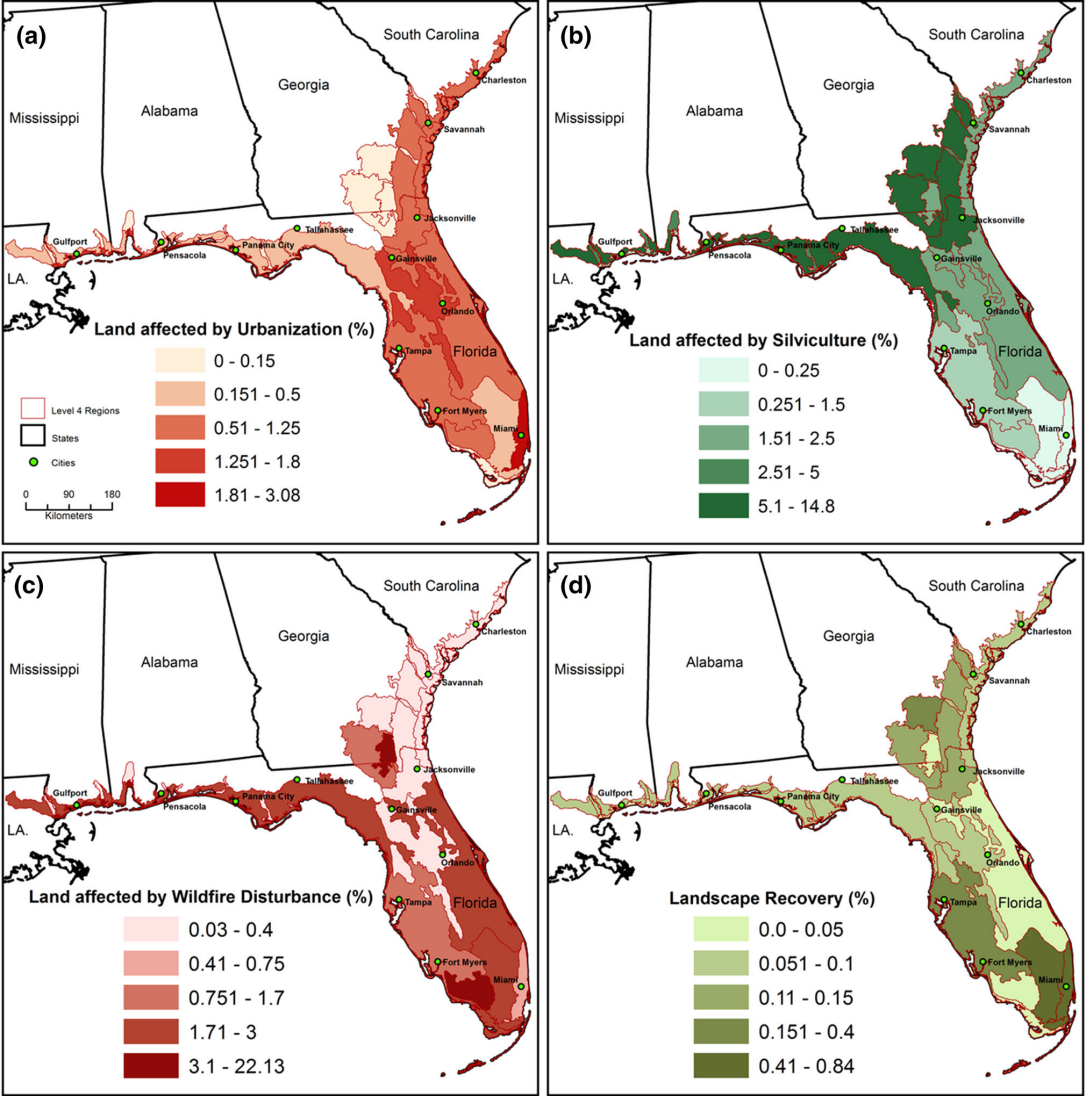
Variability of four landscape-change processes between 2001 and 2006 across the EPA level IV ecoregions, **a** urbanization, **b** total forest harvest/reforestation activity, **c** fire disturbance, and **d** total amount of landscape recovery. *Values* are expressed as a percent of ecoregion area

The drivers of urban growth prior to 2007 are well known, including rapid population growth and migration from the Midwest and Northeast, a warm year-round climate, retirement and recreation amenities, lower taxes, a burgeoning service economy, and the higher relative value of land for real estate development as opposed to agriculture and forestry uses (Mohl and Mormino 1996; Wear and Greis 2002; Auch et al. 2004; Long 2005; Montes Rojas et al. 2007; Hernández et al. 2012). Despite this, urban growth is not a major proximate cause of change in all ecoregions (Figs. 5, 7). The extent of new urban land cover is below 0.5 % (annual rate ≤0.1 %) in half of the ecoregions. These landscapes tend to have extensive corporate ownership of forest land such as in the north or have large tracts of protected or currently undevelopable land.

The estimated number of SECP residential building permits issued during the 5-year interval between 2001 and 2006 (1.11 million permits), based on county data, was nearly five times higher than during the next 5-year interval from 2007 to 2011 (230,000 permits) (U.S. Census Bureau 2014a, b), suggesting that urban growth may have declined sharply after 2006. Domestic in-migration was greatly reduced after 2006 as Florida’s housing economy collapsed during the Great Recession, although metropolitan Miami, being one of the primary gateway cities to the US for foreign immigration, helped the SECP continue to gain population and more compact multi-family housing (Frey 2010).

Land-cover replacement caused by land uses other than urban is most prevalent in the Bacon Terraces and Southwestern Florida Flatwoods, caused by a low level of gross cropland expansion (3330 ha) and substantial localized phosphate mining (11,155 ha), respectively. Cropland and pasture/hay land had some small gross increases across several ecoregions, however, twice as much agricultural land in the SECP was replaced by a combination of urbanization, expanded mining, and reservoir construction. In spite of losses due to urbanization, episodic killing freezes, and citrus diseases, Florida remained the second leading global region of orange juice production behind Brazil (Norberg 2011). New reservoir construction and residential ponds in new suburban developments contributed to net surface water increases that occurred everywhere except the Floodplains and Low Terraces. Artificial ponds constructed for esthetic and stormwater retention purposes have increased the area of surface water in other parts of the coastal plain, including southern Mississippi (Schweizer and Matlack 2014). Surface water storage is likely to continue to increase with population growth and climate variability, as long as suitable locations for reservoirs exist. The conversions to plantation silviculture (4244 ha) came primarily from agricultural land; however, it did not result in a net gain for forest cover.

#### Forest Dynamics and Recurrent Processes

The total extent of all recurrent processes, from Fig. 4, ranges greatly from a low of 0.9 % in Miami Ridge to a high of 24.0 % in the Okefenokee Swamp where fire disturbance nearly comprises the total extent of change. Land-cover change caused by the most extensive SECP recurrent processes, forest harvest and reforestation, affected more than 5 % of ecoregion extent in four northern ecoregions (Gulf Coast Flatwoods, Okefenokee Plains, Sea Island Flatwoods, and Bacon Terraces) between 2001 and 2006. The importance of intensive silviculture diminishes toward the south (Fig. 7).

The overall amount of SECP reforestation (including lands in transition to reforestation) relative to the extent of forest harvest is approximately 75 %. At the landscape scale, the measure of reforestation relative to forest harvest is higher in several northern ecoregions where intensive pine plantation silviculture is most active, most notably in the Bacon Terraces (105.5 %) and Sea Island Flatwoods (93.8 %) (Table 7). The large differences in this metric, which ranges from near 0 % to greater than 100 %, may reflect the shifting mosaic of silviculture activities that depend on decades of biomass accumulation and market timing. Very low rates of replanting, particularly in southern ecoregions, are likely not indicative of intensive plantation activity.

**Table 7:** Amount of reforestation (including areas that are ‘in transition to reforestation’) relative to forest harvest between 2001 and 2006, in percent

Ecoregions	Reforestation	Harvest	Amount of reforestation relative to forest harvest
75A Gulf Coast Flatwoods	3.7	4.8	77.5
75B Southwestern Florida Flatwoods	0.1	0.5	14.1
75C Central Florida Ridges & Uplands	0.7	1.6	42.3
75D Eastern Florida Flatwoods	0.8	1.7	47.9
75E Okefenokee Plains	6.8	8.0	84.8
75F Sea Island Flatwoods	5.9	6.3	93.8
75G Okefenokee Swamp	0.6	1.2	51.0
75H Bacon Terraces	5.2	4.9	105.5
75I Floodplains and Low Terraces	1.2	2.7	46.2
75J Sea Islands/Coastal Marsh	0.9	1.0	91.3
75K Gulf Barrier Islands & Coastal Marshes	0.1	1.2	9.8
75L Big Bend Coastal Marsh	0.1	0.2	35.6
76A Everglades	0.0	0.0	–
76B Big Cypress	0.0	0.5	0.1
76C Miami Ridge/Atlantic Coastal Strip	0.0	0.1	1.5
76D Southern Coast & Islands	0.0	0.0	–

Reforestation and harvest are based on percent of ecoregion area

Approximately 85.4 % of SECP forest harvest and reforestation activities occurred in areas with some corporate forest ownership, when summarized using US Forest Service 2007 forest ownership data (Nelson et al. 2010). The ownership data used here are at a coarser scale (250 m) than the 30-m landscape-change data and do not explicitly disclose ownership by location but do provide an indication that most forest harvest/reforestation activities are associated with corporate pine plantations. An additional 9.1 % of forest harvest/reforestation activities occurred on public lands, and approximately 5.5 % occurred on other private lands. The amount of reforestation relative to harvest is substantially higher on corporate lands than on public and other private lands (approximately 81, 39, and 58 %, respectively), indicating a higher intensity of timber management occurring on corporate pine plantations.

**Fig. 8:**
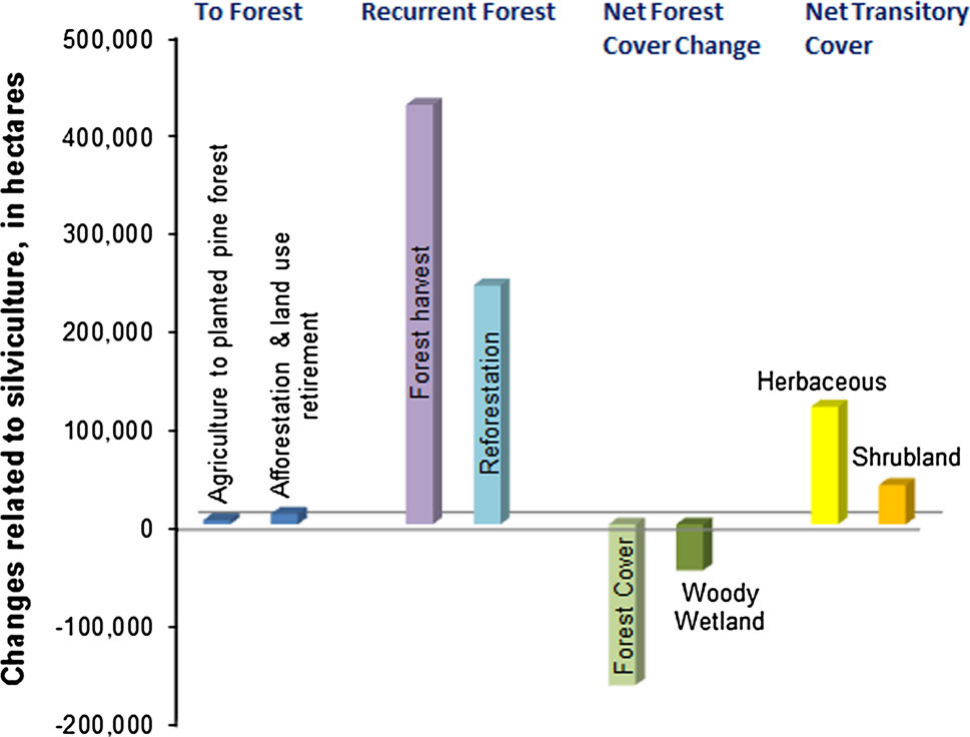
Dynamics of landscape change related to silviculture activities

Intensive silviculture activity (747,882 ha, from Table 2), which affected both evergreen forest and woody wetland, caused a nearly 184,000 ha difference between the area of harvest and reforestation (excluding areas in transition to reforestation) (Fig. 8). This accounts for a combined 1.1 % effective decline in evergreen forest and woody wetland cover, albeit as part of a recurring process with different implications than a linear conversion. By comparison, urbanization caused a 0.3 % decline in evergreen forest and woody wetland combined. Intensive silviculture also caused a net transitory increase in herbaceous and shrubland cover types, which often persist for less than a decade. Transitions to forest cover from agriculture and land retirement were relatively low, indicating that processes for acquiring new forest cover play a minor role in the region.

The total area of harvested and replanted forest cover accounted for approximately 58.3 % of all SECP processes. Because of the recurrent nature of the land use, such that harvested areas are usually replanted, this type of shifting change is not considered strictly as deforestation. However, the forest extent and other land covers are affected as the landscape transitions through vegetation stages, e.g., from forest cover to grassland and shrubland.

Fire disturbance, which affected approximately 1.8 % of the SECP, occurred in all ecoregions. However, the extent varied substantially from a low of 0.03 % in the Floodplains and Low Terraces to a high of 22.1 % in the largely protected Okefenokee Swamp (Fig. 7). Fires are naturally controlled by climate; however, humans alter the extent, intensity, and timing of fire regimes in ways that differ from a climate influence alone (Slocum et al. 2007). Less than 2 % of fire disturbance caused a noticeable decrease in forest cover.

#### Landscape Recovery Dynamics

Recovery to a semi-natural land cover from a prior land use occurred across less than 1 % of the total area in all 16 ecoregions. It is highest in the Everglades at 0.8 %, where it also represents 17.4 % of all processes of change. The Everglades has been the subject of restoration activities since the 1940s, including recent purchase of agricultural land to construct wetlands, though as much as half of the Everglades may be beyond restoration (Sklar et al. 2005). Anthropogenic changes to hydrology caused by historical drainage efforts and land use caused a drop in water table, soil subsidence, saltwater intrusion, water chemistry changes, and loss of important tree island habitat (Sklar et al. 2005; Larsen et al. 2011; McVoy et al. 2011; Willard and Bernhardt 2011). Stormwater Treatment Areas, including restored wetlands, have been created to decrease high concentrations of phosphorus from entering the protected areas of the Everglades (Maltby et al. 2009). The highly organic soils of the Everglades Agricultural Area have suffered losses from oxidation and subsidence, losing up to several centimeters a year, although the rate has slowed due to improved management practices (Wright and Hanlon 2013). In the future, soil losses in the high-value sugarcane and winter vegetable agricultural area could spur some landowners to seek alternative land uses (Snyder 2004).

Landscape recovery related to mining reclamation occurs primarily in the phosphate sites of the Southwestern Florida Flatwoods. Mining reclamation was observed in 0.3 % (7500 ha) of the ecoregion, while the extent of mining concurrently expanded by 0.5 % (11,155 ha). Reclamation of phosphate mine lands is required by Florida state law, often with topsoil replacement and revegetation projects (Brown 2005). Mining reclamation does not necessarily restore the prior land cover, such as with a progression from forest cover to mining to grassland cover, although secondary forest recovery could be a long-term result (Zipper et al. 2011). Additionally, the small amount of recovery through agricultural retirement and afforestation may be related to the Conservation Reserve Program that provides payments to return environmentally-sensitive cropland to long-term cover including trees (USDA 2006).

### Summarizing the Characteristic Processes of Landscape Change

Three general characteristics of landscape processes were explored in this study: replacement, recurrence, and recovery. Replacement processes, including urbanization and new agricultural development, are a significant part of landscape change in the SECP, though less extensive than recurrent changes. Standardized measures that differentiate urban area infill from expansion, such as explored here, may provide a useful way to compare development trajectories across large areas, and merit further examination. New agricultural development replaced forest and other land covers in various SECP landscapes, even as agriculture declined overall. Some of this land-cover replacement could be the result of agricultural displacement from elsewhere in the region (Emili and Greene 2014), suggested by concomitant agricultural losses to urban and plantation forestry during the study period, or it could be part of a typical pattern of fluctuation that results from many different individuals making site-specific decisions.

Recurrent processes that are cyclic or frequent tend to cause land-cover fluctuation as well as directional changes that complicate the analysis of land change. The forest plantation harvest and replanting cycle also causes a substantial spatial–temporal fluctuation of grassland and shrubland cover and is the dominant process of land-cover change at the regional scale. Because of the fluctuating nature of intensive cutting and regrowth cycles, the understanding of the extent and direction of forest cover change benefits from detailed information. Intensive plantation silviculture is a shifting pattern of land use that extends well beyond the area of activity captured in a snapshot. Harvested areas are generally replanted to forest rather than replaced by another land use. However, reforestation often lags harvest, which causes an effective decrease of regional forest cover and substantial landscape-scale variability. Cyclical forest harvest and regrowth is a typical process of change, and a process-based analysis across multiple time steps will further contribute to understanding the trajectory of the interconnected land-cover changes.

Landscape recovery, though a small fraction of all processes, is explicitly examined here on par with the other more dominant processes of recurrence and replacement. This effectively elevates recovery to a more conspicuous level, which is useful for understanding the overall direction of land change and issues of landscape sustainability. This type of approach is necessary to further the discussion of whether the driving forces of landscape trajectories will facilitate sustainable ecosystems and services. As such, the understanding of the various directions and processes of land-use and land-cover change is fundamental to conservation strategies. Different landscapes often undergo different trajectories, with some that are more anthropogenic and more intensive than others (Munroe et al. 2013). This research is a step toward providing a more comprehensive analysis of the spatial–temporal variability and trajectory of land-cover replacement, intensive land systems, recovery from prior land use, and landscape restoration.

Processes that feed ‘natural’ forest recovery, referred to here as afforestation, are significantly limited by current pressures on land resources, and as urbanization expands, the potential for net forest cover gain and persistence may be reduced. Only 10,640 ha of land was identified as transitioning to forest cover through agricultural retirement and afforestation. This was insufficient to counter the overall net decrease in forest cover. Some or all of this new forest cover may eventually be replaced by forest plantation activity. Historically, as eastern US forests recovered on abandoned agricultural land, there was a substantial net gain in forest cover, even as some forest clearance continued. More recently, the forest gains have slowed, and in the SECP they amount to only a small extent of the region. Forest clearance is much more extensive than forest gains from agriculture.

A total of 4244 ha of agricultural land was identified as converting to tree plantations. Intensive plantation silviculture is prevalent throughout the southeastern US and other world regions. Although plantation forests affect the extent and characteristics of forest cover, they potentially contribute to forest habitat connectivity and carbon reduction (Brockerhoff et al. 2008; Daigneault et al. 2012). Plantation forests may also restore some ecosystem services when agriculture is replaced (Benayas and Bullock 2012), although the loss of agricultural provisioning services needs to be considered as well. The increase in dense plantation forests and loss of persistent pasture and grassland can also cause a loss of habitat for some species that prefer open sites (Lymn and Temple 1991). It is unclear how an increase in transitory grassland and shrubland cover affects habitat. Plantations could also become a future stepping stone for transitioning to secondary forest ecosystem recovery (Vieira et al. 2009). In other locations, especially at the urban edge, plantation forests may instead be co-opted for housing development (Masek et al. 2011).

## Conclusions

Current measures of human pressure from land use are incomplete without including measures of silviculture and mining that are explored here (Geldmann et al. 2014). Additionally, they are incomplete without measures of human-driven landscape recovery. This analysis explored the importance of understanding the relative extent of recovery processes, which should be augmented with an improved understanding of the future capacity for landscape sustainability and recovery. Additional longer-term analysis is also needed to confirm that the extent that forest plantation land may originate from formerly persistent forest cover is small, since it may not have been detectable by this study. Subtle changes that are not typically captured without targeted remote sensing-based land-cover change analysis, such as what might occur with some ecosystem restoration projects, may also be underrepresented here.

While extensive research has been done on land-cover change in ecological regions, this study improves on previous analyses and helps illustrate the utility of a more detailed landscape framework. The variability of biophysical and human use factors within level III ecoregions is further reduced by using a landscape-scale level IV ecoregion framework. As environmental changes are occurring differently in different landscapes for different reasons, an ecoregional framework that incorporates biotic, abiotic, terrestrial, and aquatic components can facilitate environmental understanding and help in making better resource management decisions (Omernik and Griffith 2014). The more detailed level IV ecoregions provide a consistent geographic framework for analysis that can be extended to other areas of the US as the approach is refined. The AVA approach, developed for this study, provides an accessible basis for identifying and assessing the diverse causes and dynamics of change across scales.
